# Regional trends in perceptions of American Shoulder and Elbow Surgeons towards barriers to access for patients with Medicaid: limited perioperative service access, low patient engagement, and decreased reimbursement are regionally consistent obstacles

**DOI:** 10.1016/j.xrrt.2025.03.007

**Published:** 2025-04-10

**Authors:** Patrick Saunders, Abhay Mathur, Jordan Frausto, Carlos D. Ramirez, Adam Z. Khan, Brad Bushnell, Hafiz F. Kassam

**Affiliations:** a Department of Orthopedic Surgery, Hoag Orthopedic Institute, Irvine, CA, USA; b School of Medicine, University of California Riverside, Riverside, CA, USA; c School of Medicine, Des Moines University, West Des Moines, IA, USA; d Department of Orthopedic Surgery, Southern California Permanente Medical Group, Panorama City, CA, USA; e Department of Orthopedic Surgery, Harbin Clinic Orthopedics, Rome, GA, USA

**Keywords:** Insurance, Medical reimbursement, Barriers to care, Medicaid, Physician perceptions, Regional medicaid variations

## Abstract

**Background:**

Medicaid is a means-tested health insurance program for low-income adults (∼30%), children (∼40%), individuals with disabilities (15%) and some elderly patients that are dual Medicare and Medicaid eligible (15%). It is jointly funded by the federal and state governments but administered by individual states. Allowing states to oversee the administration of Medicaid has led to regional variability in eligibility criteria, types of services covered, and reimbursement. The purpose of this study was to evaluate current perceptions of shoulder and elbow surgeons surrounding practice patterns and barriers to access for patients whose primary insurance type is Medicaid, and to determine if there are notable regional variations in these opinions.

**Methods:**

This was a national, observational study that surveyed the American Shoulder and Elbow Surgeons (ASES) society membership. This 15-question survey assessed surgeon demographics, practice types, reimbursement models, as well as rates and trends of their access to patients with government-assisted insurance. Regional trends in perceived barriers to access for patients with Medicaid were specifically compared in this study.

**Results:**

A total of 257 (18.5% response rate) ASES members completed the survey. The mean year in practice for respondents was 14. The most represented region was the South (35%), followed by the Midwest (24%) and the West (20%) and Northeast (20%). Our results showed that across all regions the top three perceived barriers to access for patients with Medicaid, in varying order, remained consistent – reimbursement, low patient engagement in their care, and the patient's ability to access perioperative services. The most significant regional difference in perceived barriers to Medicaid access was due to implant reimbursement at surgeons' primary surgical facilities. In the South, nearly half (46%) of respondents viewed this as a barrier, whereas only 16% in the Northeast did.

**Conclusion:**

Among members of the ASES, the primary perceived barriers to access for patients with Medicaid remained consistent across geographic region and included reimbursement, low patient engagement in their care, and patient's ability to access perioperative services. The greatest regional disparity in perceived barriers to access for Medicaid was between the South and the Northeast in regard to implant reimbursement at surgeons' primary surgical facility. This was perceived as a barrier to access in 30% more of the Southern respondents compared to respondents from the Northeast. Further investigation into regional differences in Medicaid administration would be valuable to assess how these variances affect patient access to subspecialized shoulder and elbow care.

Medicaid is a means-tested health insurance program for low-income adults (∼30%), children (∼40%), individuals with disabilities (15%) and some elderly patients that are dual Medicare and Medicaid eligible (15%).^24^ It is jointly funded by the federal and state governments but administered by the individual states.^8^ Allowing states to oversee the administration of Medicaid, or a state equivalent, has led to variability in eligibility criteria, types of services covered, and reimbursement. As of October 2024, enrollment data from the Centers for Medicare & Medicaid Services (CMS) show 72 million people enrolled in Medicaid across the 50 states and the District of Columbia.^24^ By comparison, Medicare (a federally funded insurance payer primarily for patients older than 65 years) has 68 million enrollees as of September 2024 making Medicaid the largest federally funded health insurance program by enrollment and an important patient population to consider and understand.^22^

In 2010, the Affordable Care Act (ACA) proved to be a significant force for Medicaid expansion. Under the ACA, most states expanded coverage to include individuals earning up to 138% of the Federal poverty level beginning in 2014.^26^ Up until 2020, the costs of these additional enrollments were fully funded by the federal government. In 2020, states became responsible for 10% of the costs associated with enrollees brought on by ACA Medicaid expansion. The federal government's share of Medicaid costs varies state to state and is calculated using the Federal Medical Assistance Percentage (FMAP). FMAP is determined annually and considers a state's per capita income relative to the national average. FMAP varies from a minimum of 50% up to 76.9%.^7^

Medicaid reimbursement rates can vary significantly by region and state, especially when it comes to specialized services. This variance is largely due to the decentralized nature of the program and the allowance for state-specific Section 1115 waivers. These waivers enable states to experiment with different methods for delivering and financing Medicaid services in order to address specific challenges faced by Medicaid populations. The CMS reviews each Medicaid 1115 waiver proposal individually to assess if its objectives align with the goals of the Medicaid program. During this process, CMS examines whether the requested waivers or expenditures comply with federal policy guidelines.^3^^,^^4^ Given that these waivers must be federally budget-neutral, any increase in funding for a particular service sector necessitates a reallocation of funds from other areas within the Medicaid budget. Generally, Medicaid tends to prioritize primary care and preventive services—such as behavioral health and responses to the opioid crisis—over specialized services like orthopedics or other surgical specialties. The former are generally considered more cost-effective for promoting long-term health outcomes.

Prior studies within the orthopedic literature have demonstrated that differences in insurance type results in discrepancies in access to perioperative services, and subsequently disparity in surgical outcomes.^3^^,^^4^^,^^6^^,^^10^^,^^14^^,^^15^^,^^18^, ^19^, ^20^, ^21^^,^^25^ Recently, Kassam et al evaluated perceptions of orthopedic shoulder and elbow surgeons surrounding practice patterns and barriers to access for patients whose primary insurance type was a government assisted payor.^13^ Based on a survey distributed to the American Shoulder and Elbow Surgeons (ASES) society membership, they found that for patients with Medicare Advantage coverage, administrative burden was perceived as the largest barrier to access. However, for patients covered by Medicare and Medicaid, reimbursement was viewed as the most significant barrier by shoulder and elbow surgeons. As Medicaid administration varies on an area-specific basis, it is probable that there are regional differences in the perceptions that surgeons have relating to patients whose primary insurance is Medicaid. Whereas Kassam et al's prior study analyzed perceived barriers to access for all types of government funded insurance from the perspective of the entire cohort of respondents as a single national pool, the purpose of this study was to evaluate the current perceptions of shoulder and elbow surgeons surrounding practice patterns and barriers to access for patients whose primary insurance type is Medicaid based on geographic region of the respondents. We aimed to determine if there are any notable regional variations in these opinions, and we hypothesized that there would be observable trends based upon geographic differences in the perceived barriers to access towards patients whose primary insurance type is Medicaid.

## Methods

### Patient demographic characteristics and selection

**Figure 1:**
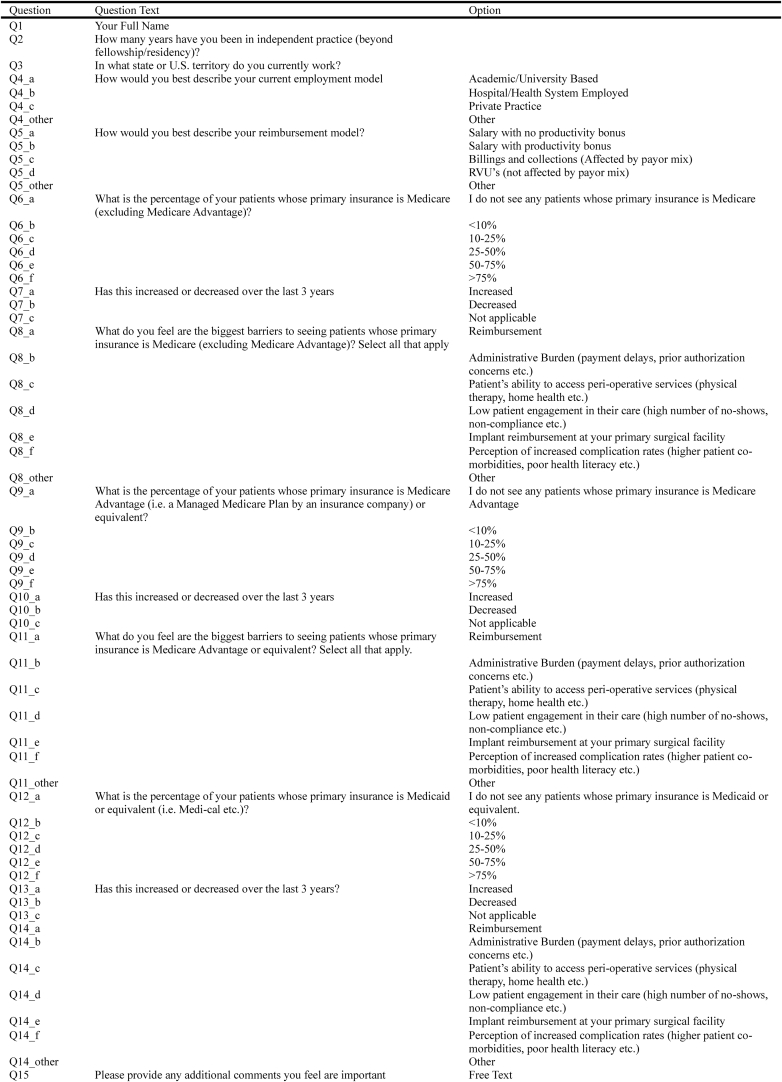
Question survey.

A 15-question, online survey (Fig. 1) for members of the Society of ASES was created to examine how surgeon demographics, employment models and payment reimbursement models affect the rate at which patients with government-assisted insurance are able to access care delivered by shoulder and elbow fellowship-trained orthopedic surgeons. Furthermore, the survey sought to identify any factors surgeons perceived as barriers to caring for these populations of patients.

The electronic survey was developed in consultation with three shoulder and elbow fellowship-trained orthopedic surgeons using SurveyMonkey (San Mateo, CA, USA) and distributed to all 1389 members of the Society of ASES between September 2024 and November 2024. Survey participation was encouraged via advertisement at the ASES annual meeting, serial email reminders, and inclusion in a random drawing for three $50 gift cards.

### Outcome metrics

Summarized data were reviewed. Data on the perceived barriers to access to specialized shoulder and elbow care for patients whose insurance payor was Medicaid were compared based on the geographic region of the respondents.

## Results

**Figure 2:**
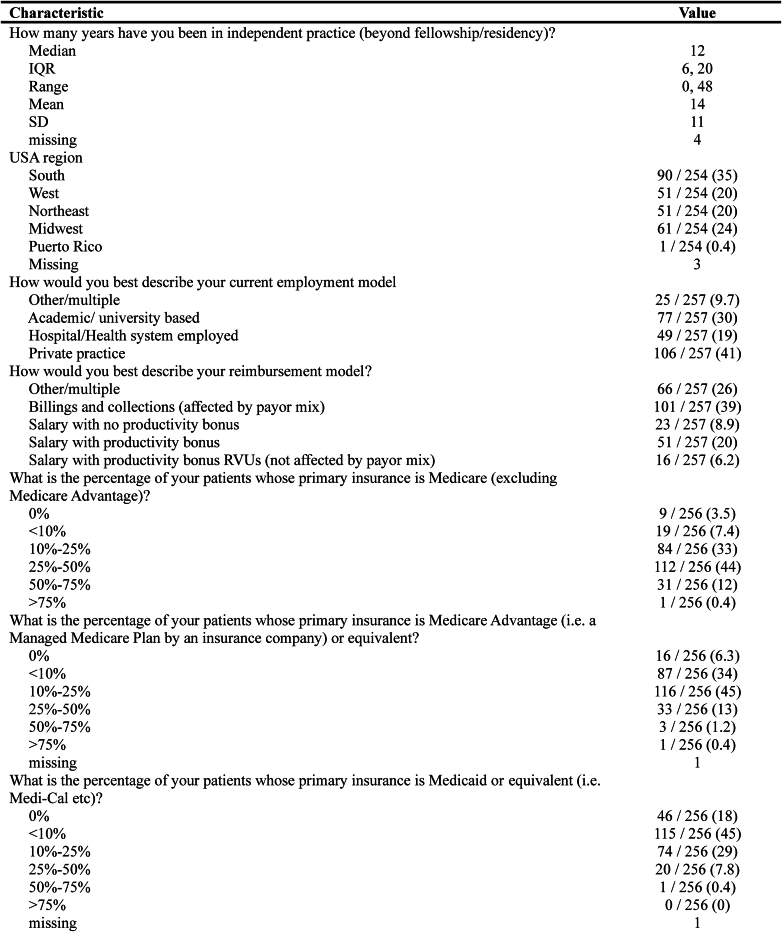
Respondent practice demographic analysis (N = 257). *IQR*, interquartile range; *SD*, standard deviation; *RVU*, relative value unit.

A total of 257 ASES members completed the survey (response rate 18.5%). Respondent demographics including employment type, reimbursement model, years in practice, geographic region, and percentage of patients seen with each type of government-assisted insurance plan are summarized in Figure 2.

### Demographics

**Figure 3:**
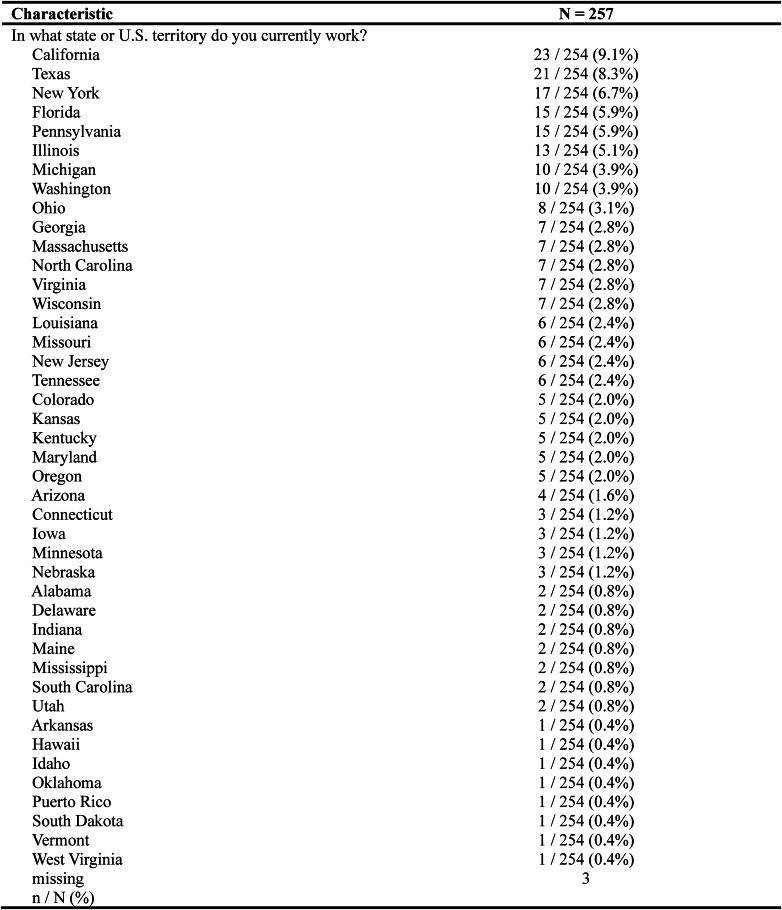
Respondent geographic analysis.

The mean years in practice for respondents was 14 (range 0-48). The most represented region was the South (35%), followed by the Midwest (24%) and the West (20%) and Northeast (20%) (Fig. 3). One respondent was from Puerto Rico. The most common employment model was private practice (41%) followed by academic or university (30%), hospital or health system employed (19%), and other (9.7%). Overall, 39% of respondents indicated that their reimbursement model was based on billings and collections and affected by payor mix. In contrast, 20% described their reimbursement model as salaried with a productivity bonus, 8.9% were salaried with no productivity bonus, and 6.2% stated they were salaried with a productivity bonus that was not affected by payor mix. Finally, 26% of respondents indicated their model was a combination of the above.

### Patient population makeup

Forty-five percent of respondents indicated that Medicaid-insured patients made up less than 10% of their practice population, and 29% stated that 10%-25% of their patient population had Medicaid as their primary insurance. However, 18% of respondents did not see any Medicaid-insured patients. There was a trend toward surgeons not taking Medicaid with more years in practice: 6% (3 of 50) for <5 years in practice, 14.5% (9 of 62) for 5-10 years in practice, 21.3% (17 of 80) for 10-20 years in practice, and 26.2% (16 of 61) for >20 years in practice.

### Perceived barriers to access

**Figure 4:**
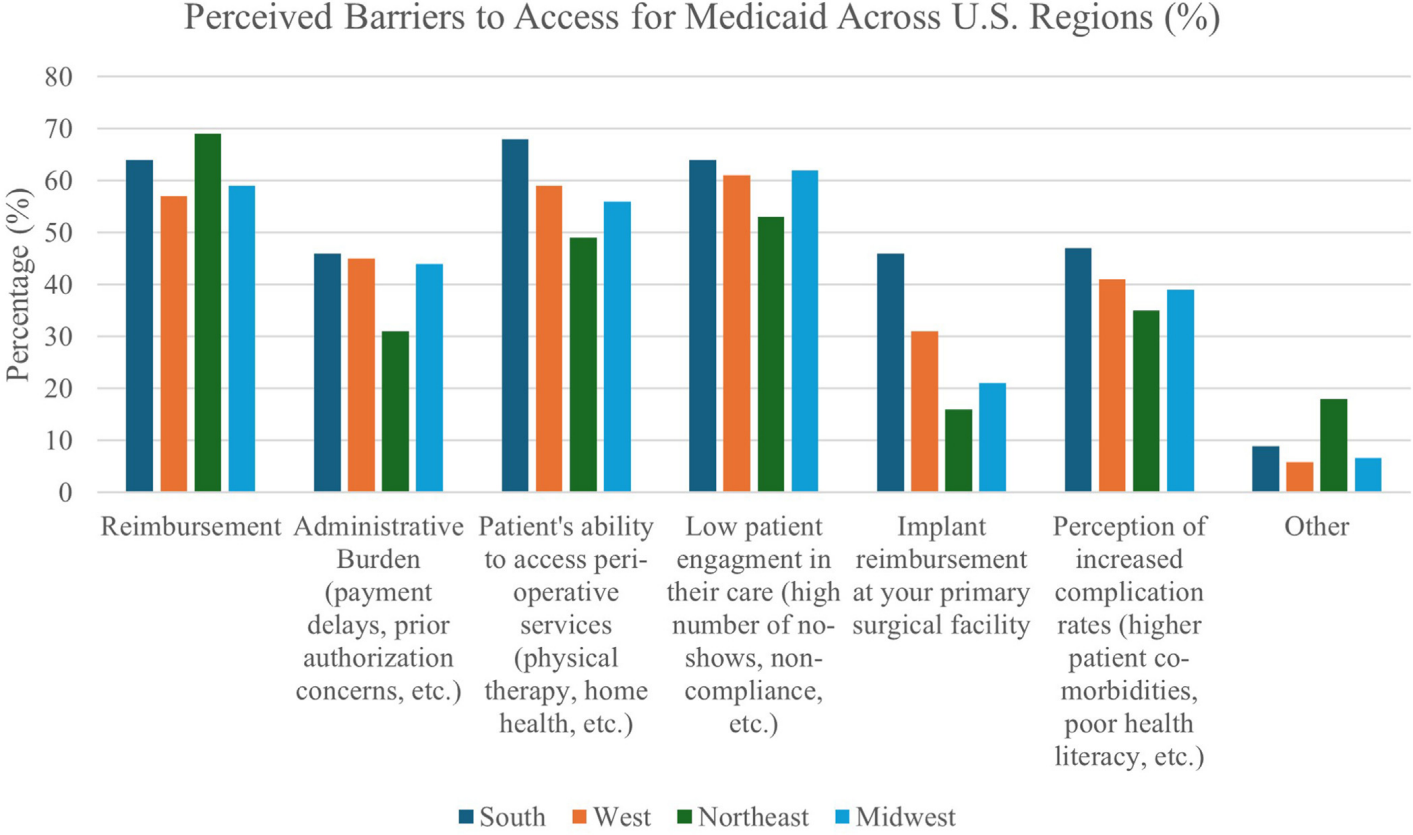
Perceived barriers to access for medicaid across US regions (%).

The most common perceived barriers for Medicaid patients across the nation, when results from all regions were pooled together, were relatively evenly distributed between reimbursement (62%), low patient engagement in their care (61%), and patient's ability to access perioperative services (60%). Respondent results to perceived barriers to access for patients with Medicaid insurance based on geographic region of the United States are summarized in Figure 4. The most common perceived barriers in the Northeast were reimbursement (69%) followed by low patient engagement in their care (53%) and then patient's ability to access perioperative services (49%). The most common perceived barriers in the South were patient's ability to access perioperative services (68%) followed by low patient engagement in their care (64%) and reimbursement (64%). In the Midwest, the most common perceived barriers were low patient engagement in their care (62%), followed by reimbursement (59%) and then patient's ability to access perioperative services (56%). Finally, in the West the most common perceived barriers were low patient engagement in their care (61%), followed by patient's ability to access perioperative services (59%) and then reimbursement (57%). Implant reimbursement at surgeons' primary surgical facility was perceived as a barrier to access in providing care for Medicaid patients among 46% of respondents in the South, whereas only 16% of respondents from the Northeast perceived this as a barrier.

## Discussion

As the American health-care system continues to change, concern among health-care providers has developed relating to increasing health-care costs in conjunction with an increasing segment of the population that relies on government funded programs for insurance coverage. A recent study by Kassam et al explores the sentiment and views of subspecialized orthopedic shoulder and elbow surgeons towards government payors to identify perceived barriers to government-assisted programs.^13^ Their results showed that for patients with Medicare Advantage administrative burden was perceived as the largest barrier to access, whereas for patients covered by Medicare and Medicaid, reimbursement was viewed as the most significant barrier by shoulder and elbow surgeons. Unlike Medicare and Medicare Advantage, Medicaid administration is overseen by each individual state. This leads to potential variability in eligibility criteria, types of services covered, and reimbursement on a state-by-state basis. While the perception of surgeons regarding barriers to access for patients with Medicare and Medicare Advantage likely does not differ based on geographic region due to the uniform federal administration of these plans, the same may not hold true for perceived barriers relating to Medicaid due to the aforementioned system of state specific oversight and administration. This study specifically assessed regional trends in how orthopedic shoulder and elbow surgeons perceive barriers to access for patients with Medicaid.

Our results showed that across all regions of country (Northeast, South, Midwest, and West) the top three perceived barriers to access for patients with Medicaid, in varying order, remained consistent – reimbursement, the patient's ability to access perioperative services, and low patient engagement in their care. Trends have shown consistent, significant, decreases in Medicare reimbursement rates for physicians for quite some time now.^1^^,^^5^^,^^17^ Shoulder and elbow surgeons have been particularly affected by these decreases. In their work investigating temporal and geographic trends in Medicare reimbursement of primary and revision shoulder arthroplasty, Testa et al showed the average reimbursement for all shoulder arthroplasty procedures decreased by 35.5% from 2000 to 2020.^23^ Historically, reimbursement from Medicaid has been even lower than Medicare, with some data showing less than two thirds the reimbursement rate.^2^^,^^12^ Given these circumstances, it comes as no surprise that reimbursement was perceived as one of the chief barriers to access among respondents across all regions. While there may be some regional variations in the reimbursement rates, concern stemming from consistent downward trends in reimbursement from government funded beneficiaries is pervasive. Likewise, the perception of patients' ability to access perioperative services and low patient engagement in their care as prominent barriers to access for Medicaid patients supersedes any regional variation in Medicaid administration. Given that Medicaid consists of the most socioeconomically disadvantaged populations in each state, it is predictable that low patient engagement and access to perioperative services are viewed as a consistent barrier regardless of regional differences among respondents. Poor health literacy, housing instability, lack of transportation, and inconsistent means of communication are all common challenges faced by large segments of the Medicaid population. These factors significantly hamper a patient's ability to access perioperative services and dissuade them from engaging in their own care, regardless of which state they reside.^16^ Our results reflect these pervasive challenges faced by Medicaid patients across state lines.

Our data showed that the greatest regional disparity in perceived barriers to access for Medicaid was between the South and the Northeast in regard to implant reimbursement at surgeons' primary surgical facility. This was perceived as a barrier to access in providing care among 46% of respondents in the South, whereas only 16% of respondents from the Northeast perceived this as a barrier. Gupta et al investigated trends in reimbursement for all billable total joint replacement procedures using the Medicare Part B database from 2013 to 2021.^9^ They found that total shoulder arthroplasties had the greatest increase in beneficiaries (124.42%) and greatest decline in Medicare reimbursement (−51.23%) over the time period.^9^ When stratified by region, the Northeast had the greatest mean Medicare reimbursement and the South had the greatest total beneficiaries.^9^ While these results pertain to Medicare as opposed Medicaid, they could be considered in the context of overall government beneficiaries as a portion of shoulder surgeons' practice populations. With the South having the highest amount of total Medicare beneficiaries, this region is more impacted by government reimbursement in general, which includes Medicaid. As reimbursement rates from government-funded plans have been universally decreasing, Southern providers likely perceive the impact of all aspects of government reimbursement in a greater manner, including reimbursement for implants. Conversely, Northeast providers, who receive the highest rates of reimbursement, and have a lower percentage of their overall income derived from government reimbursement, likely do not perceive reimbursement for implants as an equally impactful barrier to access for patients with Medicaid.

Interestingly, Haddad et al performed a retrospective review to determine if open payments made by industry arthroplasty companies to physicians and hospital systems were significantly affected by implant type and geographic variation.^11^ In contrast to the aforementioned findings showing the Northeast had the greatest rates of government beneficiary reimbursement, Haddad's results demonstrated that reverse total shoulder implants were associated with significantly higher open payments in the South and West regions compared to the Northeast.^11^ While the underlying cause for this is likely complex and multifactorial, it may be in part related to the regional variations seen in reimbursement rates for government beneficiaries. Due to the lower relative reimbursement rates seen in the South compared to the Northeast, providers in this region may be less incentivized to perform shoulder arthroplasties for Medicaid patients as it may not be as financially beneficial or efficient for them or their practice. It may be the case that industry arthroplasty companies are motivated to contribute larger open payments to physicians in the South to offset the inferior governmental reimbursement rates, and thus make it more appealing for providers to perform arthroplasties. These regional differences seen in open payments made by industry arthroplasty companies may be a result of the same underlying mechanisms which drive our results showing the regional difference in perceived barriers to access between the South and the Northeast relating to implant reimbursement.

This study is not without limitations. Our data represent surveyed opinions and approximations of a collection of subspecialized shoulder and elbow surgeons. Bias may be present in our data given that these are surveyed judgements rather than definitive insurance claims data. However, given that ASES members are typically high-volume shoulder surgeons, we feel these assessments provide a valuable origin to understand and assess perceived barriers to care for patients with Medicaid. Moreover, many of these survey questions aimed to gauge surgeon opinion, which can occasionally be considered to play a more significant role than true raw data when it comes to accepting different insurance contracts. These data are a gathered from a subset of specialized orthopedic surgeons, thus may not be as generalizable to all surgical subspecialities or nonsurgical medical practices. In addition, the response rate among the surveyed population was very low (18.5%), which limits the generalizability of the results even among the specific subset of shoulder and elbow surgeons. Finally, there was not an equal distribution of respondents from each state and not all states had respondents, therefore the results may be skewed to represent the opinions of surgeons within certain states rather than the designated regions as a whole.

## Conclusion

Among members of the ASES the primary perceived barriers to access for patients with Medicaid remained consistent across geographic region and included reimbursement, low patient engagement in their care, and patient's ability to access perioperative services. The greatest regional disparity in perceived barriers to access for Medicaid was between the South and the Northeast in regard to implant reimbursement at surgeons' primary surgical facility. This was perceived as a barrier to access in providing care among 46% of respondents in the South, whereas only 16% of respondents from the Northeast perceived this as a barrier. Further investigation into regional differences in Medicaid administration would be valuable to assess how these variances affect patient access to subspecialized shoulder and elbow care.

## Disclaimer:

Funding: No funding was disclosed by the authors.

Conflicts of interest: Adam Z. Khan MD receives research support funding from DePuy, Johnson and Johnson, and Stryker. This research support is for studies not related to this paper. This author has been compensated by DJ Orthopedics for speaker presentations. Brad Bushnell MD receives research support funding from Smith and Nephew. This research support is for studies not related to this paper. This author has been compensated by Smith and Nephew for speaker presentations as well as consulting. The other authors, their immediate families, and any research foundation with which they are affiliated have not received any financial payments or other benefits from any commercial entity related to the subject of this article.
